# Optimization of the roasting conditions to lower acrylamide content and improve the nutrient composition and antioxidant properties of *Coffea arabica*

**DOI:** 10.1371/journal.pone.0237265

**Published:** 2020-08-25

**Authors:** Huluager Endeshaw, Abera Belay

**Affiliations:** Department of Food Science and Applied Nutrition, Addis Ababa Science and Technology University, Addis Ababa, Ethiopia; Institute for Biological Research "S. Stanković", University of Belgrade, SERBIA

## Abstract

Roasting is the most common method of processing coffee. During roasting, aromatic compounds are generated due to various reactions, which are important for developing color, flavor and aroma. Acrylamide is an undesirable carcinogenic substance that is metabolically activated and formed during the coffee roasting process. *Coffea arabica* was first found in Ethiopia, and Ethiopia can produce a large volume of coffee. The major coffee-producing areas in Ethiopia are Hararghe, Sidama, Gimbi/Nekemte, Yergachefe and Limu. The primary purpose of this study was to quantify the acrylamide contents of brewed and roasted coffee collected from street coffee sellers and industrial processors found in Addis Ababa, Ethiopia, and optimize the roasting conditions for Sidama coffee. The acrylamide contents were determined by HPLC using a DAD at 210 nm, the antioxidant property were examined using a UV–spectrophotometer, and moisture and nutrient composition of coffee was determined using the method described by the AOAC (Association of Official Analytical Chemists). The roasting temperature and time were optimized based on the acrylamide content, nutritional composition and antioxidant property of the coffee using central composite design. The roasting temperature and time significantly affected (p<0.05) the acrylamide level, nutritional composition and antioxidant property of the coffee. The acrylamide contents of street and industrial processed powdered coffee were 346 ±19 to 701±38μg/kg and 442±14 to 906±7μg/kg, respectively. Brewed coffee from street vendors and industrial processing had acrylamide contents of 25±2 to 49±1μg/L and 63±2 to 89±4μg/L, respectively. The EC_50_ values for scavenging radicals for the optimized coffee ranged from 171±0 to 111±4 μg/L. The optimal roasting temperature and time were 190°C and 6 minutes, at this temperature and time the acrylamide content decreased, and the antioxidant and nutritional compositions of the coffee improved.

## Introduction

Coffee is the most frequently consumed beverage around the world [1]. It has a good fragrance, taste, health benefits and caffeine content, which make it a highly acceptable beverage throughout the world. *Coffea arabica* is cultivated in Ethiopia, and a large volume of coffee can be produced in this country. According to Chauhan et al. [2], Ethiopia’s cool climatic conditions and rainfall create favorable conditions for coffee cultivation.

Food processing is an important factor in improving the nutritional qualities, microbiological safety, and sensory qualities while removing some compounds with potential negative health impacts [3]. Roasting is the most well-known processing method and is important for coffee preparation. During roasting, aromatic compounds are generated due to different reactions, such as the Maillard reaction, Strecker degradation, the degradation of sugars and the breakdown of amino acids, which are important for developing color, flavor and aroma [1]. Acrylamide is a thermal processing contaminant that is produced in the Maillard reaction, a series of nonenzymatic reactions between reducing sugars and free amino acids [4,5]. The products of fat breakdown, mainly acrolein and acrylic acid, have also been found to be precursors for acrylamide formation [5,6]. Acrylamide is not found in raw and unprocessed food items. It is formed when food containing certain precursors are thermally processed at temperatures of 120°C or above, such as by frying, baking, roasting, grilling and toasting [7,8].

According to Morales et al. [9], compounds with health benefits, such as antioxidants, can be generated during roasting. However, heat-induced toxic food contaminants with carcinogenic and genotoxic properties such as heterocyclic amines, acrylamide and furfurals can also be generated during roasting, especially if the processing temperature and time are not tightly controlled [10]. The International Agency for Research on Cancer (IARC) has classified acrylamide as a probable human carcinogen [11]. Roasting coffee at very high temperatures also causes the degradation of antioxidant compounds, such as caffeine, chlorogenic acid (CGA) and Maillard reaction products (melanoidins) [12].

The European Commission (EC) developed recommendations and references in 2013 to control the acrylamide content of food products. The reference values for roasted and instant coffee were 450 μg/kg and 900 μg/kg, respectively [13]. The newly revised EC recommendations for acrylamide in roasted and instant coffee are 400 and 850 μg/kg, respectively [14]. Dibaba et al. [15] reported that the level of acrylamide in ‘*Keribo’*, a traditional Ethiopian fermented beverage, was as high as 3440 μg/kg. However, the content of acrylamide and the optimal time and temperature for coffee roasting were not investigated for Ethiopian coffee. In this study, the acrylamide contents of brewed and roasted coffee samples that were prepared by street coffee brewers and coffee processors was examined, and the roasting temperature and time were optimized for Sidama coffee. Accordingly, the acrylamide content, antioxidant properties and nutritional composition were determined.

## Materials and methods

### Materials

Coffee samples were collected based on Madihah et al. [1]. Street and industrially processed brewed and powdered coffee were obtained from local markets in Addis Ababa sub-cities (Arada, Akaki-Kality, Nifas Silk-Lafto and Kirkose); using randomized lottery sampling methods; and Sidama coffee was used in the optimization of roasting temperature and time. Sidama coffee is one of the majorly produced and highly exported coffee type in Ethiopia, and was collected from Ethiopian Coffee Quality Inspection and Certification Center (ECQICC), Addis Ababa.

### Experimental design

Response surface design through central composite design (CCD) used to optimize the roasting time and temperature with 14 runs. The roasting temperature and time ranged from 180°C to 200°C and 4 to 8 minutes, respectively. A quadratic model was used to find the optimal roasting conditions.

### Roasting of the coffee beans

The coffee beans were roasted based on Madihah et al. [1], with some modifications. Accordingly, a Probat roaster machine (PROBAT, Gimborn Maschinenfabrik GmbH, Germany, Emmerich am Rhein) was used for the roasting process. The machine was first heated to 170°C, and 100 g of green coffee beans put into the roasting cylinder. The coffee beans were roasted for different combinations of roasting time and temperature using CCD (170 to 200°C for 4 to 8 minutes). The roasted beans were tipped out into a cooling tray and allowed to cool for an average of 4 minutes by blowing cold air over the cooling plate. Visual inspection was used to separate the roasted coffee beans based on color using an AGTRON- Specialty Coffee Association of America (SCAA, 1995). The roasted coffee beans were cooled, finely ground in a coffee grinder (Mahlkoning grinder, Hemro Manufacturing, Germany, Hamburg) to a screen size of 3.5 (0.30 mm), packed in a sealed plastic bag and stored at 4°C until analysis.

### Acrylamide determination

#### Sample extraction

The roasted coffee samples were extracted with water using SPE cleanup for acrylamide analysis, following the method described by Oroian et al. [16]. Accordingly, 2.5 g of coffee powder and 10 ml of brewed coffee were weighed in a 50 ml centrifuge tube. Ten milliliters of water and 5 ml of n-hexane were added, and the sample was mixed for 5 minutes and then centrifuged (Universal 320, Andreas Hettich GmbH & Co. KG, Tuttlingen, Germany) at 2509 g (4000 rpm) for 5 minutes. The upper hexane layer was then discarded, and 1 ml of Carrez I solution and 1 ml of Carrez II solution were added. Then, the mixture was centrifuged at 2509 g (4000 rpm) for 5 minutes. The clear intermediate layer was again purified by adding 1 ml of Carrez I solution and 1 ml of Carrez II. The sample was mixed vigorously for 5 minutes and then centrifuged at 2509 g (4000 rpm) for 5 minutes. The clear layer was transferred to an SPE column previously conditioned with 1 ml of methanol and 1 ml of water. The extract was passed through the cartridge. The first five drops were discarded, and 4 ml of filtrate was collected and concentrated to dryness using nitrogen gas. The residue was reconstituted in 2 ml of water. It was then filtered through a 0.20 μm filter disk and transferred to autosampler vials for HPLC-DAD determination of the acrylamide.

#### Acrylamide analysis

The acrylamide contents of different roasted coffee samples were determined by HPLC (Agilent 1260 infinity HPLC, Agilent technologies, USA, Minnesota). The separation of the compounds was achieved using an Agilent ZORBAX Eclipse Plus column) (Agilent ZORBAX Eclipse plus column 4.6*100mm, 3.5μm, Agilent technologies, USA, Minnesota.). The data were processed with Agilent Open Lab CDS software (Agilent Open Lab CDS software, Agilent technologies, USA, Minnesota). The flow rate was 0.50 ml/ minutes, and the column oven was fixed at 30°C. The total run time was 15 minutes with 10 minutes for acquisition and 5 minutes for postrun. The injection volume was 20 μl. The mobile phase was 99% HPLC-grade water and 0.1% acetic acid solution. Acrylamide was detected using a diode array detector set at 210 nm [17]. The linearity of the calibration curve was evaluated by plotting peak area as a function of analyte concentration and assessing the goodness of fit of the linear regression [18]. Sensitivity of the method was determined with the Limit of Detection (LOD) 5 μg/l and Limit of Quantification (LOQ) 20.40 μg/kg [18]. Relative standard deviation percent (RSD%) was evaluated by analyzing replicates of acrylamide and was obtained 2.63% [19].

### Nutrient composition determination

The moisture content was determined using AOAC [19] method 925.09. AOAC [19] method 941.12 was used to determine the ash content. AOAC [19] method 4.5.01 was used to determine the total crude fat content. The protein content of roasted coffee beans was determined using AOAC [19] method 979.09. The crude fiber content of the roasted coffee powder was determined using AOAC [19] method 920.169. AOAC [19] method 985.29 was used to determine the percentage of total carbohydrates. The gross energy was determined based on Kim et al. [20]. Accordingly, the fat, carbohydrate and protein contents were calculated by conversion factors.

### Antioxidant determination

#### Sample extraction

The extraction of the coffee samples for antioxidant determination was based on Abdeltaif [21]. Accordingly, 5 g of coffee was weighed into a flask, and 50 ml of methanol was added. The sample was shaken for 24 h and then filtered through fat-free filter paper (Whatman filter paper). Again, 50 ml of methanol was added, and the mixture was shaken for 2 h and then filtered. The filtrate was transferred into a round bottom flask and then concentrated to a rotary evaporator (RE-2000A, Germany RE 501 5L Rotovap, N/A, Canada, Oshawa). The sample was reconstituted in methanol and stored in a refrigerator at 4°C until analysis.

#### Total polyphenols

The total phenol content of roasted coffee was analyzed using a UV-vis spectrometer (Lambda 950Uv-Vis/NIR spectrophotometer, EUROIMMUN a PerkinElmer company, Germany, Lübeck) according to Singleton and Rossi [22]. Briefly, 1 ml of Folin-Ciocalteu phenol reagent was added to the solution. After 5 minutes, 10 ml of 7% Na_2_CO_3_ was added, and the total volume was brought to 25 ml by deionized water. Then, the sample was incubated for 90 minutes, and the absorbance was measured at 765 nm. The TPC is expressed as mg of GAE/g of extract and was determined using the following equation.

$$TPC=\frac{C*V}{M}*D$$ Model 1

where C: gallic acid equivalent concentration obtained from the calibration curve (mg/ml); V: volume of stock solution of extract (ml); M: dry weight of extract in the stock solution (g); TPC: total phenol content (mg of GAE/g dry extract); and D: dilution factor.

#### Flavonoids

The flavonoid content in the roasted coffee was analyzed using a UV-vis spectrometer (Lambda 950Uv-Vis/NIR spectrophotometer, EUROIMMUN a PerkinElmer company, Germany, Lübeck) [23]. The samples and quercetin standards were at different concentrations. After 5 minutes, methanol and 1 ml of 10% aluminum chloride were added. Then, the absorbance was measured at 415 nm, and the results are expressed as quercetin equivalents (mg quercetin/g dried extract) and were calculated using the following equation:

$$FC=\frac{C*V}{M}*D$$ Model 2

where C: quercetin equivalent concentration obtained from the calibration curve (mg/ml); V: volume of stock solution of the extract (ml); M: dry weight of extract in the stock solution (g); FC: flavonoid content expressed as (mg of CE/g dry extract); and D: dilution factor.

#### Radical scavenging activity

The radical scavenging activity (DPPH) of the roasted coffee was analyzed using a UV-vis spectrometer (Lambda 950Uv-Vis/NIR spectrophotometer, EUROIMMUN a PerkinElmer company, Germany, Lübeck) by Abdeltaif et al. [21]. The sample solution and ascorbic acid standards were prepared at different concentrations, and methanol was added. Then, 4 ml of DPPH was added, and the mixtures were placed in the dark for 90 minutes. The absorbance was measured at 517 nm, and the inhibition of DPPH free radical was calculated using the following equation:

$$I\%=\frac{Ac-As}{Ac}*100$$ Model 3

where Ac is the absorbance of the control reaction and As is the absorbance of the test compound.

#### Ferric reducing power

The FRAP (ferric reducing antioxidant power) of the roasted coffee was analyzed by using a UV-vis spectrometer (Lambda 950Uv/NIR spectrophotometer, EUROIMMUN a PerkinElmer company, Germany, Lübeck) according to Benzie et al. [24], with some modifications. Fresh FRAP reagent (2.5 ml of TPTZ (10 mM in 40 mM HCl), 25 ml of acetate buffer and 2.5 ml of FeCl_3_6H_2_O) was prepared and incubated at 37°C for 15 minutes. The test samples and ascorbic acid standards were taken at different concentrations, and methanol was added. Then, 3 ml of the freshly prepared FRAP reagent was added, and the mixtures were incubated for 30 minutes. The absorbance was measured at 593 nm.

#### EC_50_ (Effective concentration)

EC_50_ (Effective concentration), the amount of sample necessary to decrease the absorbance of DPPH by 50%, computed based on the method stated by Alexander et al. [25]. EC_50_ was calculated by considering the dose-response curves obtained by plotting the percentage of inhibition versus concentration.

### Statistical analysis

All tests were performed in duplicate. Response surface design (RSD) was used to optimize the roasting time and temperature, and the data were analyzed using SPSS version 20. One-way ANOVA was used to determine the effect of roasting on the acrylamide content, the nutritional composition and the antioxidant properties of roasted coffee. Multiple comparison tests using the least significant difference (LSD, p = 0.05) technique were applied to compare the means of each parameter between different coffee samples.

## Results and discussion

### Acrylamide content of powdered coffee

The acrylamide contents in powdered coffee from street vendors and industrially processed coffee ranged from 346±19 to 906±0 μg/kg (Table 1). A significant difference (p<0.05) was observed among the acrylamide contents of powdered and brewed coffee among street vendors and industrially processed coffee due to the roasting and brewing conditions. This is in line with the report of Pastoriza et al. [26] that during dark roasting, acrylamide reacts with melanoides to form the color and flavor of coffee, which causes the content of acrylamide to decrease.

**Table 1:** Acrylamide contents of brewed and roasted coffee collected from street vendors and coffee processors presented as the mean ±sd.

Treatment	Acrylamide content	
	Powder (μg/kg)	Brewed (μg/L)
SA	346±19^a^	25±2^a^
SB	408±0^b^	37±7^b^
SC	701±0^e^	49±1^c^
SD	435±1^b,c^	41±5^b,c^
PA	477±0^c^	71±6^d,e^
PB	538±1^d^	77±3^e^
PC	906±0^f^	89±4^f^
PD	442±1^b,c^	63±2^d^

Treatments with the same letter across columns are not significantly different (p>0.05).

SA = street coffee sample from sub city A; PA = industrially processed coffee sample from sub city A; SB = street coffee sample from sub city B; PB = industrially processed coffee sample from sub city B; SC = street coffee sample from sub city C; PC = industrially processed coffee sample from sub city C; SD = street coffee sample from sub city D; PD = industrially processed coffee sample from sub city D.

Bagdonaite et al. [27] reported that the coffee species had an impact on the level of acrylamide formed during roasting, and the acrylamide contents in 12 roasted coffees ranged from 299 to 762 μg/kg, which was in agreement with this finding. The findings of this study for street coffee samples (from sub-city A, B, C, D) and industrially processed coffee samples (from sub-city A, B, C, D) (Table 1) was in agreement with the acrylamide content reported by Surma et al. [28] (17±1 to 776±8 μg/kg). Bertuzzi et al. [29] reported that the mean acrylamide level of 66 coffee samples collected from Italy was 465 μg/kg. The acrylamide contents of 291 coffee samples from Europe ranged from 79 μg/kg to 1188 μg/kg [30]. The acrylamide content found in this study agreed with the acrylamide contents of Italian and European processed coffees. The maximum limit of acrylamide for roasted coffee in the EU is 400 μg/kg [14]. The mean acrylamide content in the coffee samples in this study (SB, SC, SD, PA, PB, PC and PD) (Table 1) was higher than the maximum limit set by EU [14]; however, the content in SA was below the limit. Lachenmeier et al. [31] reported that the acrylamide content in pure roasted coffee reduced over the years. None of the samples exceeded the EU benchmark. Samples, which have considerably above the EU benchmark, could be a mix with cereals [31]. Hence, further study needs to conduct on the purity of the roasted/brewed samples collected from Addis Ababa.

### Acrylamide content of brewed coffee

The acrylamide contents in brewed coffee samples from street vendors and industrial processing were 25 to 89μg/L (Table 1). A significant difference (p<0.05) was observed between the acrylamide contents of brewed coffee from street vendors and industrial processed coffee due to the coffee preparation and roasting conditions. This finding was in agreement with the report of Sirot et al. [32] (37 μg/L), Alves et al. [33] (1.7 to 75 μg/L) and Mesías & Morales [8] (7.7 to 40 μg/L).

### Effect of roasting temperature and time on the acrylamide content of Sidama Coffee

The acrylamide content (mean±sd) of optimally roasted Sidama coffee is given in Table 2. The acrylamide contents of optimally roasted Sidama coffee were 202 and 2549μg/kg for very light and medium dark roasted coffee, respectively. There was a significant difference (p<0.05) between the roasted coffee samples due to the roasting conditions. This was similar to EFSA, Contam [34] reported that the roasting temperature and time were the major factors affecting the formation of acrylamide in coffee. The report of Claeys et al. [35] (170 to 2522 μg/kg) was in agreement with this finding. However, the report of Wenzl & Anklam [30] on European coffee (79 μg/kg to 1188 μg/kg) reported a higher acrylamide content for Sidama coffee. This could be due to the coffee species, roasting conditions and duration of storage. According to Bagdonaite et al. [27], the coffee roasting conditions affect the formation of acrylamide. The r^2^ value and adjusted coefficient of acrylamide for optimally roasted coffee were 0.55 and 0.24, respectively. According to Zhang et al. [36], for good fit, the r^2^ should be at least 0.80. The r^2^ for these response variables was less than 0.80. This indicated that the estimated quadratic model was not good. This was related to the unstable formation of acrylamide. According to Madihah et al. [1], the content of acrylamide formed was not stable. It forms at high temperatures and is also destroyed at high temperatures (^o^C).

**Table 2:** Optimization of the acrylamide content in roasted Sidama coffee (mean ±sd, n = 14).

Temperature (°C)	Time (minute)	Acrylamide (μg/kg)	Run number
175.86	5.50	202±14^a^	12
180.00	7.00	281±10^b^	1
180.00	4.00	263±18^b^	6
190.00	5.50	360±0^c^	3
190.00	5.50	359±0^c^	5
190.00	5.50	358±0^c^	7
190.00	5.50	359±0^c^	8
190.00	7.62	2549±6^g^	9
190.00	5.50	359±0^c^	10
190.00	5.50	357±3 ^c^	11
190.00	3.38	344±15^c^	13
200.00	4.00	1988±7 ^f^	2
200.00	7.00	577±15^e^	4
204.14	5.50	511±34^d^	14

Means with different letters across columns are significantly different (p<0.05).

Independent variable -1 0 1

Temperature (°C) 180 190 200

Time (minutes) 4 5.5 8

**Fig 1:**
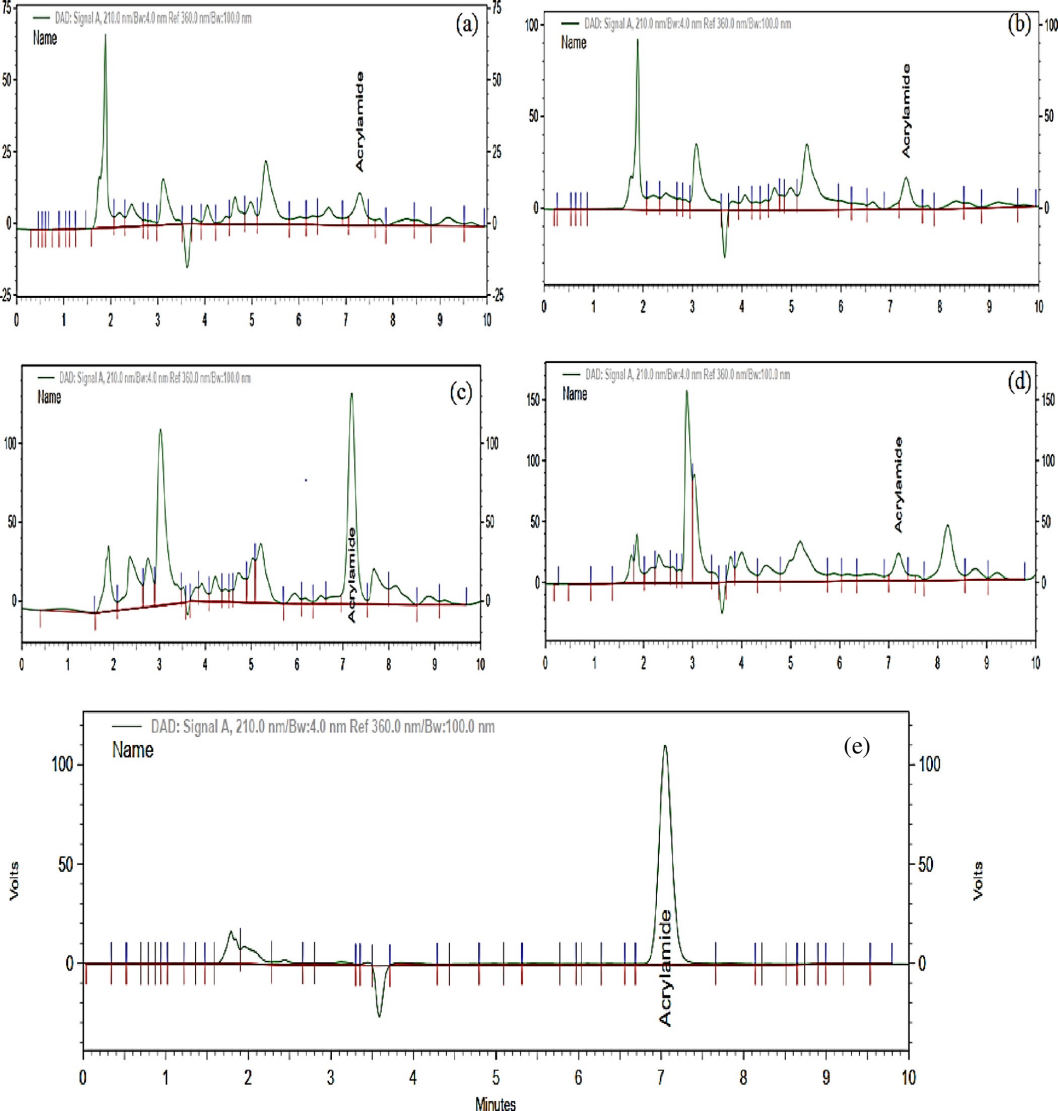
Chromatogram representation of acrylamide for different roasting condition. a) light roasted coffee; b) medium roasted coffee; c) medium dark roasted coffee; d) dark roasted coffee; e) standard.

The chromatogram of acrylamide under different roasting conditions are presented in Fig 1. This study revealed that acrylamide content increases with increasing roasting temperature until it reaches a maximum after which it rapidly decreases. In this study, medium dark roasted coffees contained more acrylamide than light and dark roasted coffees. This was in line with Lantz et al. [37], who found that the acrylamide content increased during the middle phase of roasting and then rapidly decreased.

**Fig 2:**
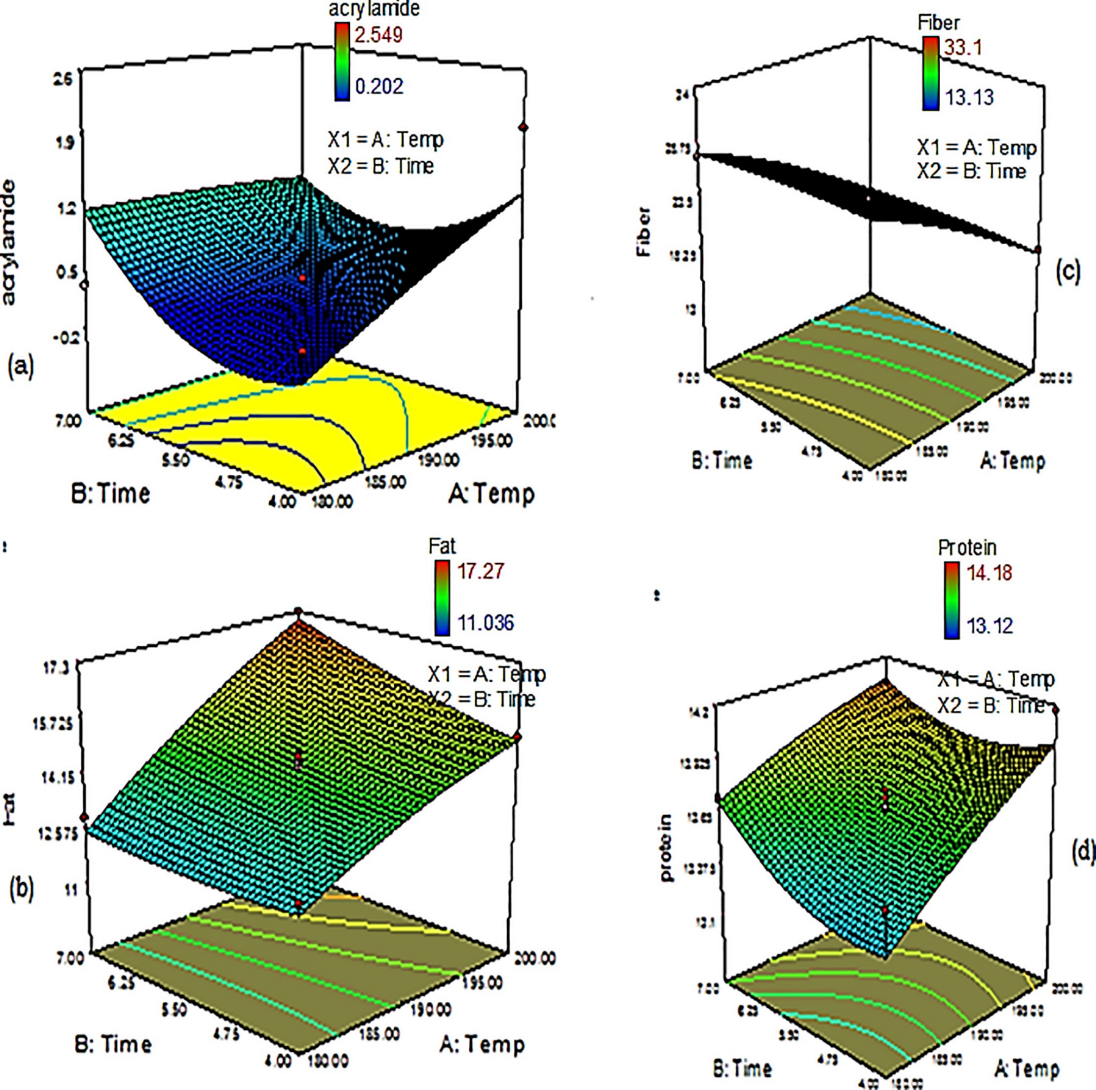
3D plot of a) acrylamide b) fat c) fiber d) protein content of optimized roasted Sidama coffee.

The acrylamide contents in very light, light, light-medium, medium-light, and medium roasted coffees were below the limit set by the European Commission (400 μg/kg) [14]. However, the contents in medium-dark, dark-medium, dark, and very dark roasted coffees were higher than 400 μg/kg. The presence of acrylamide, in association with the color type of the roasted coffee (light to dark), appears in contrast to the report of Lachenmeier et al. [31], though this study equally reported that acrylamide content declined, with an increase at a higher extreme temperature. For the reason, that degree of roasting certainly yield different categories of color (very light to very dark), and the variation could be the capacity of coffee beans to possess different color levels during roasting.​ At a similar temperature and time of roasting, coffee color varies from region to region, and roasting does not yield consistent results. This is mostly governed by size, weight, and moisture content of coffee beans [38], and coffee bean variety, reducing sugars, sucrose level, available free amino acids, and pH [39]. As a result, color alone could not be a reliable assessment for roasting level [40], which demands further study. The optimal roasting time and temperature for Sidama coffee were 190°C for 6 minutes, as this resulted in a low acrylamide (336 μg/kg) content (Fig 2A). In relative term, this was in agreement with the report of Chung [41] (182 ^o^C and 7 minutes).

### Nutrient composition of Sidama coffee

#### Moisture

The moisture contents of Sidama coffee at different roasting temperatures and times ranged from 0.45 to 3.59% (Table 3). The moisture content in very light roasted coffee (3.59±0) was higher than that in medium (2.23±0) and dark roasted coffee (0.91±0). Accordingly, there was a significant difference (p<0.05) in moisture contents between treatments due to the roasting conditions. The moisture contents of medium, medium-dark (1.43±0.) and dark-medium (1.21±0) roasted coffee were in agreement with the reports of Franca et al. [42] on Brazilian roasted coffee (1.5%). On the other hand, very dark roasted Sidama coffee (0.45%) had a lower water content than that of Brazilian dark roasted coffee [42]. The moisture contents of very light, light and light-medium roasted coffee agreed with the reports of Tawfik & El Bader [43].

**Table 3:** Nutrient compositions of roasted Sidama coffee (mean ± sd).

Run	Temp	Time (minutes)	Moisture	Fat	Protein	Ash	Fiber	Carbohydrate	Total energy
	(°C)		(%)	(%)	(%)	(%)	(%)	(%)	(%)
1	190	5.5	2.14±40^d^	14.54±0^c^	13.73±0^,b^	3.93±0^a,b^	23.82±0 ^c^	41.85±0^c^	353.13±5^c^
2	200	7	0.91±0^b^	16.29±0^e^	13.55±0^a,b^	4.2±0^b^	15.09±0^a^	48.97±0^e^	405.58±0^f^
3	180	7	2.59±0^e,f^	12.94±0^b^	14.04±0^b^	3.78±0^a,b^	27.45±1^d,e^	39.19±1^b^	329.44±7^b^
4	190	5.5	2.08±0^d^	14.42±0^c^	13.76±0^a,b^	3.95±0^a,b^	23.95±0^c^	41.83±0^c^	352.19±1^c^
5	190	5.5	2.11±0^d^	14.51±0^c^	13.73±0^a,b^	3.93±0^a,b^	23.85±0^c^	41.89±0^c^	353±1^c^
6	180	4	2.83±0^f^	12.93±0^b^	14.16±0^b^	3.67±0^a,b^	30.12±2^e^	36.28±1^a^	318.14±13^b^
7	200	4	1.21±0^b,c^	15.23±1^c,d^	13.54±0^a,b^	3.94±0^a,b^	18.32±1^b^	47.76±0^d,e^	382.32±13^e^
8	175.9	5.5	3.59±0^g^	11.04±0^a^	14.18±0^b^	3.32±0^a^	33.10±1^f^	34.77±1^a^	295.12±1^a^
9	204.1	5.5	0.45±0^a^	17.28±0^d,e^	13.12±0^a^	4.38±0^b^	13.13±0^a^	52.64±0^f^	409.63±2^f^
10	190	5.5	2.09±0^d^	14.47±0^c^	13.75±0^a,b^	3.94±0^a,b^	23.9±0^c^	41.86±0^c^	352.58±0^c^
11	190	5.5	2.23±0^d^	14.70±0^c^	13.67±0^a,b^	3.88±0^a,b^	23.61±0^c^	41.95±0^c^	354.49±0^c^
12	190	5.5	2.03±0^d^	14.36±0^c^	13.79±0^a,b^	3.98±0^a,b^	24.05±0^c^	41.8±0^c^	351.64±1^c^
13	190	7.62	1.43±0^c^	15.07±0^c,d^	13.68±0^a,b^	4.02±0^a,b^	19.63±1^c^	46.1785±1^d^	375.08±7^e^
14	190	3.38	2.30±0^d,e^	13.94±0^b,c^	13.93±1^a,b^	3.73±0^a,b^	25.09±1^c,d^	41.01±0^c^	345.24±4^c^

Means across columns with the same letters are not significantly different (p>0.05).

#### Fat

The fat contents of roasted Sidama coffee ranged from 11.04 to 17.28% (Table 3). The fat content in dark roasted coffee (16.29±0%) was higher than that in medium (14.47±0%) and light roasted coffees (12.93±0%). Accordingly, the fat contents in the samples varied significantly (p<0.05) due to the roasting conditions. This was in agreement with the findings of Liu et al. [44] that the fat content of coffee beans increases with roasting temperature due to the degradation of carbohydrates and the evaporation of volatile chemicals. In addition, the oil also released to the outer surface of the bean during roasting. Tawfik & El Bader [43] reported that the mean fat contents of roasted Harar and Berry coffee were 11.83 and 12.30%, respectively. The fat content of roasted Sidama coffee was higher than that of Harar and Berry coffee beans. According to Franca et al. [42], healthy mature coffee beans have a higher oil content than defective beans.

Accordingly, Sidama coffee can be classified as a good quality coffee. The optimal temperature and time for roasting to achieve better fat formation were 190°C at 6 minutes, which resulted in 14.42% fat (Fig 2B).

#### Protein

The protein contents of the roasted Sidama coffee samples ranged from 13.12 to 14.18% (Table 3). The protein content of very dark roasted Sidama coffee was significantly different (p<0.05) from that of light-medium, light and very light roasted coffee. There were no significant differences (p>0.05) among the protein contents of very light, light, light-medium, medium-light, medium, medium-dark, dark-medium and dark roasted coffee. The protein contents reported by Belitz et al. [45] ranged from 9–12%, which are lower than the protein contents of roasted Sidama coffee. Farah et al. [46] reported that the protein contents of green and roasted coffee ranged from 9–16% and 11–16.5%, respectively. The protein content in roasted Sidama coffee was similar to the result reported by Farah [46]. The optimal temperature and time for achieving the highest protein content were 190°C and 6 minutes, respectively, which afforded a protein content of 13.69% (Fig 2D).

#### Ash

The ash contents of roasted Sidama coffee samples range from 3.32 to 4.38% (Table 3). The ash content of very light roasted coffee was significantly different (p<0.05) from those of dark and very dark roasted coffees. According to Bicho [47], the total mineral content in coffee beans is approximately 4%. The ash content of roasted Sidama coffee is consistent with that report. However, the ash content of roasted Sidama coffee was lower than the ash content reported by Oliveira et al. [48] (4.8% to 5.8%).

#### Fiber

The fiber contents in roasted Sidama coffee ranged from 13.13 to 33.1% (Table 3). The fiber content of dark roasted coffee (15.09±0%) was lower than that of medium (23.95±0%) and light roasted coffee (30.12±2%), and a significant variation (p<0.05) was found between very light roasted coffee and light, light-medium, medium-light, medium, medium-dark, dark-medium, dark and very dark roasted coffees. The fiber contents in coffee reported by Ismail et al. [49] were 15.45 to 21.51%, which is in agreement with the content found for roasted Sidama coffee. This value was also similar to that reported by Martinez-Saez et al. [50] (19% to 23%). The optimal temperature and time for achieving a high fiber content were 190°C and 6 minutes, respectively, which afforded a fiber content of 22.60%.

#### Total carbohydrate

The carbohydrate contents of the roasted Sidama coffee samples were 34.77 to 52.64% (Table 3). The carbohydrate content in dark roasted coffee (48.97±0%) was higher than that of medium (41.85±0%) and light roasted coffee (36.28±1%). Accordingly, light-medium and very dark roasted coffee had significantly different contents (p<0.05) from those of very light, light, medium-light, medium, medium-dark, dark-medium and dark roasted coffee due to the roasting conditions. Nogaim et al. [51] reported the carbohydrate contents of 70 coffee samples from Yemen (7.92 ± 0 to 35.64 ± 2), and their results were in agreement with the carbohydrate contents of Sidama coffee. However, the carbohydrate contents of the roasted Sidama coffees were lower than those reported by Franca et al. [42] (61.49 to 62.41%). The optimal roasting temperature and time to achieve the highest carbohydrate content was 190°C for 6 minutes, which afforded a carbohydrate content of 42.92%.

#### Total energy

The total energy values of the roasted Sidama coffee samples were 295.12 to 409.63 kcal (Table 3). The roasting temperature and time were found to influence the total energy of the coffee. The total energy of the dark roasted coffee (405.58±0 kcal) was higher than that of the medium (353.13±5 kcal) and light roasted coffees (318.14±13 kcal). Accordingly, very light roasted coffee significantly differed (p<0.05) from the light, light-medium, medium-light, medium, medium-dark, dark-medium, dark and very dark roasted coffees in total energy content. The optimal temperature and time for achieving a high total energy content were 190°C and 6 minutes, respectively, which afforded a total energy content of 344.6 kcal.

### Antioxidant properties of Sidama coffee

#### Total phenolic compounds

The effects of roasting temperature and time on the antioxidant properties of roasted coffee are presented in Table 4. The total phenol contents in roasted Sidama coffee ranged from 26 to 63 mg GAE/g (Table 4). Light-medium roasted coffee (63±5 mg GAE/g) contained more phenolic compounds than light (45±4 mg GAE/g) and dark roasted coffees (33±1 mg GAE/g). Accordingly, there were significant differences (p<0.05) among the phenol contents in the roasted Sidama coffees due to roasting time and temperature. The content of phenolic compounds in coffee depend on coffee variety, origin, degree of roasting and method of beverage preparation [52]. In this study, the content of phenolic compounds decreased with increasing temperature. Tamilmani [53] reported that roasting temperature causes chlorogenic acid degradation, which consequently reduces the polyphenol content in the coffee. Hečimović et al. [54] reported the phenol contents of cherry and minas coffee varieties (21mg GAE/g to 42mg GAE/g), which were in agreement with this finding. The phenolic contents of Sidama coffee were also in agreement with Perez-Martinez et al. [55] (37 to 55 mg GAE/g).

**Table 4:** Antioxidant contents and activities of Sidama coffee within different roasting conditions (mean ± sd).

Run number	Temp (°C)	Time (min)	Phenol (mg GAE/100 g)	Flavonoid (mg Qu/100 g)	DPPH (μg/ml)	FRAP (mg/g)	EC_50_ (μg/ml)
1	180	4	45±4^d,e^	63±9^c,d^	84±0^e^	7±1^b^	77±0^b^
2	200	7	33±1^a,b^	29±5^a^	68±2^b^	5±1^a,b^	102±3^f^
3	180	7	63±5^f^	73±7^d^	90±2^f^	6±1^a,b^	71±0^a^
4	190	5.5	38±2^b,c,d^	53±0^b,c^	79±0^d^	6±1^a,b^	85±0^c^
5	190	5.5	38±0^b,c,d^	53±0^b,c^	78±0^d^	6±1^a,b^	85±0^c^
6	200	4	35±0^b,c^	33±1^a^	71±0^b,c^	5±0^a,b^	99±0^f^
7	190	5.5	38±0^b,c,d^	54±0^b,c^	79±0^d^	6±2^a,b^	85±0^c^
8	190	7.62	33±1^a,b^	33±0^a^	73±1^c^	5±1^a,b^	93±0^d^
9	190	5.5	38±0^b,c,d^	53±0^b,c^	78±0^d^	6±1^a,b^	84±0^c^
10	190	3.38	51±5^e^	64±7^cd^	81±0d^e^	6±1^ab^	81±0^bc^
11	190	5.5	38.±0^b,c,d^	54±0^b,c^	79±0^d^	6±0^a,b^	85±0^c^
12	175.86	5.5	41±1^c,d^	49±0^b^	83±0^e^	6±1^a,b^	78±0^b^
13	190	5.5	38±0^b,c,d^	54.±0^b,c^	79±0^d^	6±1^a,b^	85±0^c^
14	204.14	5.5	26±0^a^	26±7^a^	64±2^a^	5±1^a^	111±4^g^

Means with different letters are significantly different (p<0.05).

#### Total flavonoid content

The flavonoid contents of the roasted Sidama coffee samples ranged from 26 to 73 mg QE/g (Table 4). The flavonoid content in light-medium roasted coffee (73±7 mg QE/g) was higher than those of very light (49±0 mg QE/g) and dark roasted coffee (29±5 mg QE/g). Accordingly, there were significant differences (p<0.05) among the flavonoid contents of roasted Sidama coffee due to roasting time and temperature. Very dark, dark, dark-medium and medium dark roasted coffee had significantly different (p<0.05) contents from those of very light, light, light-medium, medium-light, and medium roasted coffee. Additionally, the flavonoid content of light medium-roasted coffee was significantly different (p<0.05) from those of medium and very light roasted coffee. This finding was similar to that of Cho et al. [56], who reported that the flavonoid content was high in light roasted coffee and decreased with increasing temperature. The flavonoid contents of Sidama coffee were also in agreement with the results reported by Kim et al. [23] (22 ±0 to 67.19 ± 0 mg QE/g).

#### Radical scavenging activity

The percent inhibition for roasted Sidama coffee ranged from 64 to 90 μg/ml (Table 4). A significant difference (p<0.05) was observed among the inhibitory activities of roasted Sidama coffees due to the roasting conditions. The radical scavenging activities of very dark, dark and light-medium roasted coffees were significantly different (p<0.05) from those of the dark-medium, medium-dark, very light, light, medium-light and medium coffees. Odžaković et al. [57] stated that the antioxidant activity of coffee decreased during roasting, and the higher antioxidant activity was obtained in medium-roasted coffee compared with dark-roasted coffee, which was in agreement with this study.

**Fig 3:**
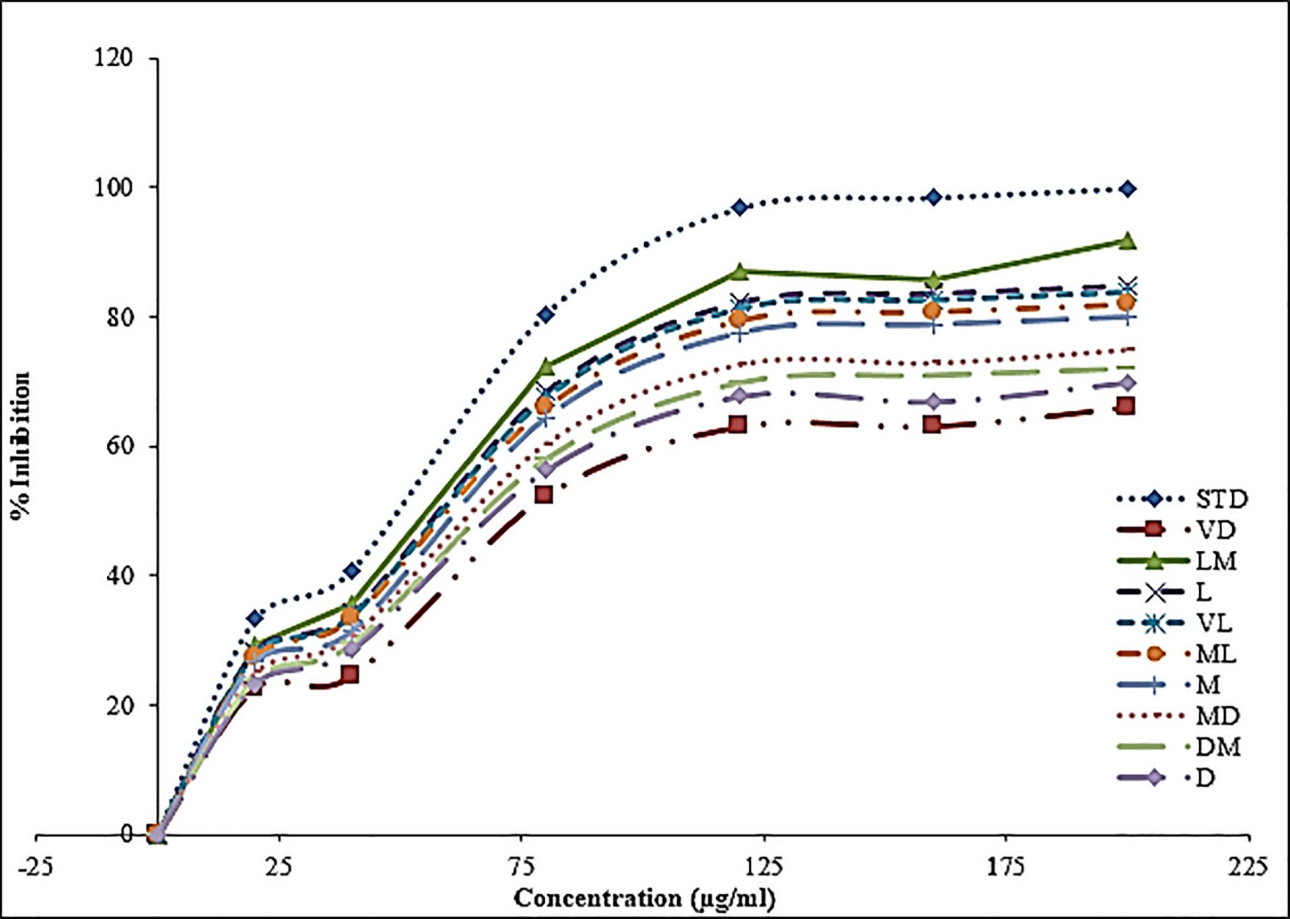
Antioxidant scavenging capacity of Sidama coffee: LM = light medium; DM = medium light; M = medium; D = dark; L = light; MD = medium dark; VL = very light; ML = medium light and VD = very dark.

A graphical representation of % inhibition for Sidama coffees roasted under different conditions is presented in Fig 3. The percent inhibition and roasting temperature are inversely related. The percent inhibition decreased with temperature. Additionally, Fig 2A shows the direct relation between % inhibition and concentration of sample.

#### Ferric reducing power

The ferric reducing activities of roasted Sidama coffee ranged from 5 to 7 mg/g (Table 4). The ferric reducing activity of light roasted coffee (7±1 mg/g) was higher than those of medium (6±1 mg/g) and dark roasted coffees (5±1 mg/g). Likewise, the ferric reducing activities vary significantly (p<0.05) due to the roasting conditions. The ferric reducing powers of the very dark and dark roasted coffee were significantly different (p<0.05) from that of light roasted coffee.

The ferric reducing activity of Sidama coffee decreased as the roasting temperature increased. This finding was similar to Cho et al. [56]; accordingly, the roasting conditions of the coffee had a significant effect on the FRAP value of the coffee. The FRAP value decreased with increasing roasting temperature due to the thermal degradation of phenolic compounds. The FRAP values of the roasted Sidama coffee samples were similar to those reported by Gebeyehu and Bikila [58] (6 ± 0 to 9 ± 0 mg/g).

**Fig 4:**
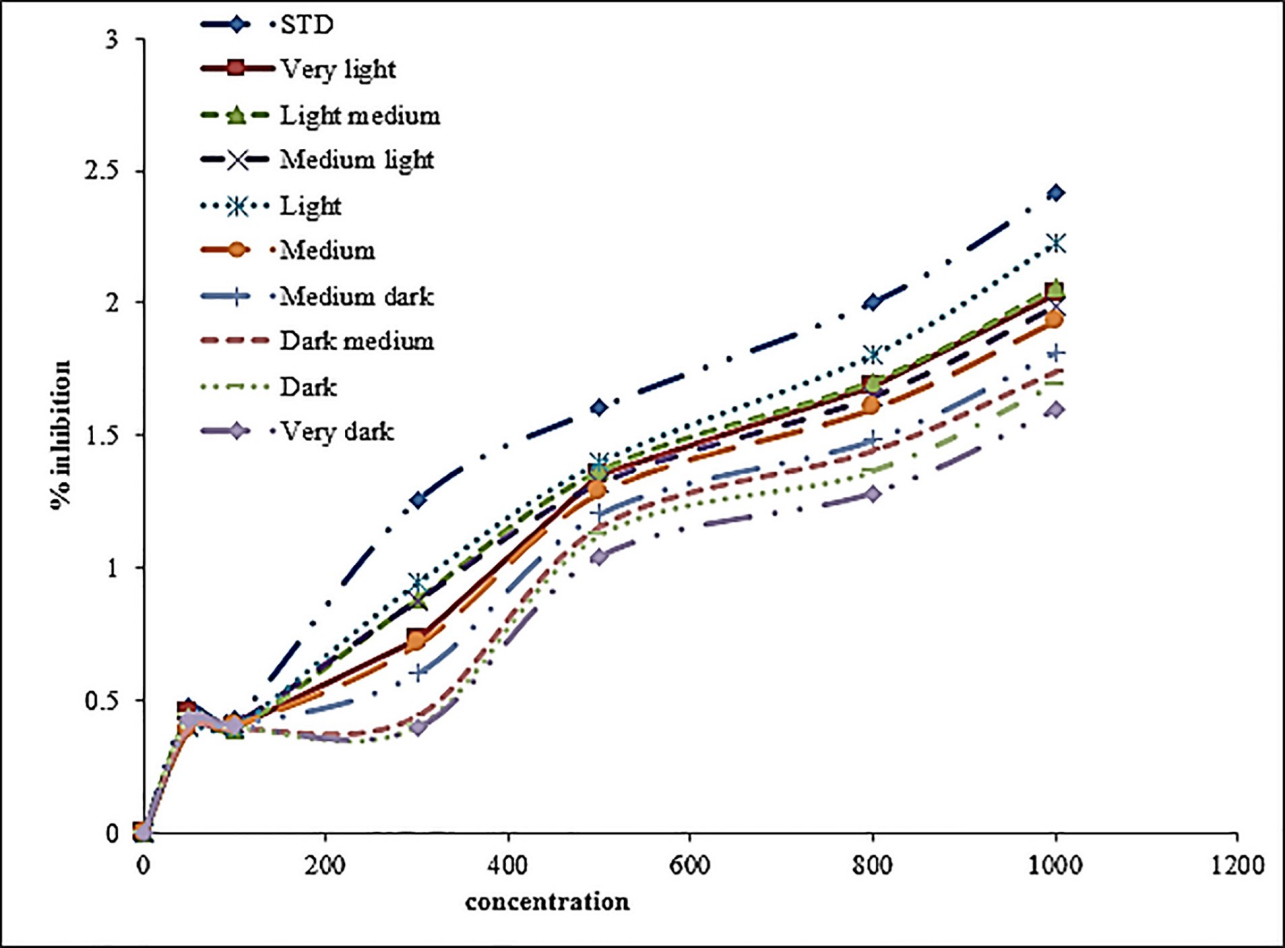
Ferric reducing power of roasting Sidama coffee.

The effect of roasting on the ferric reducing activity of roasted Sidama coffee is presented in Fig 4. Accordingly, ferric reducing power had an inverse relationship with the roasting temperature. Light roasted coffee had a higher ferric reducing power than dark roasted coffee. Fig 2B also shows that the ferric reducing power was directly related to the concentration of the sample. The ferric reducing power increases with increasing concentration.

#### Effective concentration (EC_50_)

The EC_50_ (effective concentration) values of the roasted Sidama coffee samples were 71 to 111 μg/ml (Table 4). The EC_50_ value of light-medium roasted coffee was higher than that of medium, light and dark roasted coffee. The EC_50_ values of the roasted Sidama coffees vary significantly (p<0.05) due to the roasting conditions. The values of light-medium, medium, medium-dark and very dark roasted Sidama coffees were significantly different (p<0.05) from those of dark, dark-medium, very light, light, and medium-light roasted coffee. This was in line with the report of Delgado-Andrade et al. [59] that the EC_50_ value decreased due to the formation of melanoidins. The content of melanoidins increased with increasing temperature, which was responsible for the reduction in the scavenging activity of the coffee.

## Conclusions

The acrylamide contents of coffee samples collected from street vendors and industrial processors were higher than the EU standards. The acrylamide contents in the coffee samples from street vendors were lower than those of industrially processed coffee due to the roasting conditions. Based on our optimization of the roasting conditions of Sidama coffee, the highest acrylamide content was achieved in medium-dark roasted coffee (190°C for 7 minutes), and the lowest was in very light roasted coffee (175.86°C for 5.5 minutes). The roasting time and temperature had an effect on the antioxidant content in the coffee. An increase in roasting decreases antioxidant properties. Accordingly, light-medium roasted coffee had a relatively high antioxidant activity. Roasting also had a significant effect on the composition of the coffee. The moisture, protein, and fiber contents of the coffee decreased as the roasting temperature increased. However, the carbohydrate, fat, ash, and total energy contents increased with roasting temperature. The optimal roasting temperature for Sidama coffee was 190°C for 6 minutes, at which point the acrylamide content decreased and the antioxidant content of coffee improved.

## Supporting information

S1 File (DOCX)
