# Supplementary material for: Optimization of the roasting conditions to lower acrylamide content and improve the nutrient composition and antioxidant properties of Coffea arabica
Source: PLoS One. 2020 Aug 25;15(8):e0237265. doi: 10.1371/journal.pone.0237265 (PMC7447024; doi:10.1371/journal.pone.0237265)
Supplement: S1 File — (DOCX) [file pone.0237265.s001.docx]

**Data used for Tables and Figures**

**Data Used for Tables**

**Data Used for Table 1. Acrylamide contents of brewed and roasted coffee collected from street vendors and coffee processors presented as the mean ±sd.**

|  | Acrylamide content | |
| --- | --- | --- |
| Treatment | Powder (µg/kg) | Brewed (µg/L) |
| SA | 332.4245 | 23.849 |
|  | 359.9514 | 27.11425 |
| SB | 405.3107 | 42.25075 |
|  | 410.8577 | 31.88475 |
| SC | 674.4432 | 50.21325 |
|  | 728.6714 | 47.8235 |
| SD | 423.5926 | 45.36625 |
|  | 448.2423 | 37.25875 |
| PA | 456.8778 | 76.36125 |
|  | 497.8165 | 67.11525 |
| PB | 547.8564 | 74.69375 |
|  | 529.8888 | 79.585 |
| PC | 912.3591 | 86.0515 |
|  | 901.4505 | 92.6955 |
| PD | 453.293 | 64.97 |
|  | 432.4916 | 62.06475 |

**Data Used for Table 2. Optimization of the acrylamide content in roasted Sidama coffee**

| Run no | Temperature  (^o^C) | Time  (minute) | Acrylamide  (µg/kg) |
| --- | --- | --- | --- |
|  |  |  |  |
| 1 | 180 | 7 | 274.2721 |
|  |  |  | 288.6521 |
| 2 | 200 | 4 | 1993.747 |
|  |  |  | 1982.889 |
| 3 | 190 | 5.5 | 359.467 |
|  |  |  | 360.554 |
| 4 | 200 | 7 | 588.9927 |
|  |  |  | 566.5862 |
| 5 | 190 | 5.5 | 359.098 |
|  |  |  | 359.25 |
| 6 | 180 | 4 | 276.6976 |
|  |  |  | 250.8241 |
| 7 | 190 | 5.5 | 358.987 |
|  |  |  | 358.655 |
| 8 | 190 | 5.5 | 359.3022 |
|  |  |  | 360.2644 |
| 9 | 190 | 7.62 | 2554.377 |
|  |  |  | 2545.533 |
| 10 | 190 | 5.5 | 358.987 |
|  |  |  | 358.655 |
| 11 | 190 | 5.5 | 360.281 |
|  |  |  | 355.016 |
| 12 | 175.86 | 5.5 | 212.774 |
|  |  |  | 192.5875 |
| 13 | 190 | 3.38 | 333.2604 |
|  |  |  | 355.1484 |
| 14 | 204.14 | 5.5 | 487.6892 |
|  |  |  | 536.2156 |

**Data Used for Table 3. Nutrient compositions of roasted Sidama coffee.**

| Temp (^o^C) | Time (min) | Moisture | Fat | protein | Ash | Fiber | Carbohydrate | Total energy |
| --- | --- | --- | --- | --- | --- | --- | --- | --- |
| 190 | 5.5 | 2.41 | 14.951 | 13.562 | 3.797 | 23.23 | 42.05 | 357.007 |
|  |  | 1.877 | 14.12 | 13.887 | 4.058 | 24.4 | 41.658 | 349.26 |
| 200 | 7 | 0.837 | 16.41 | 13.593 | 4.137 | 14.95 | 49.421 | 405.614 |
|  |  | 0.978 | 16.18 | 13.512 | 4.257 | 15.239 | 48.526 | 405.544 |
| 180 | 7 | 2.371 | 12.497 | 14.55 | 3.574 | 28.625 | 38.383 | 324.205 |
|  |  | 2.818 | 13.391 | 13.537 | 3.978 | 26.276 | 40 | 334.667 |
| 190 | 5.5 | 2.14 | 14.53 | 13.72 | 3.92 | 23.81 | 41.88 | 353.17 |
|  |  | 2.01 | 14.32 | 13.8 | 3.99 | 24.1 | 41.78 | 351.2 |
| 190 | 5.5 | 2.21 | 14.63 | 13.68 | 3.89 | 23.66 | 41.93 | 354.1 |
|  |  | 2 | 14.38 | 13.78 | 3.97 | 24.03 | 41.84 | 351.9 |
| 180 | 4 | 2.79 | 12.31 | 14.231 | 3.294 | 32.149 | 35.226 | 308.618 |
|  |  | 2.875 | 13.557 | 14.087 | 4.055 | 28.1 | 37.326 | 327.665 |
| 200 | 4 | 1.156 | 14.017 | 13.887 | 3.677 | 19.56 | 47.703 | 372.513 |
|  |  | 1.257 | 16.451 | 13.193 | 4.193 | 17.08 | 47.826 | 392.135 |
| 175.86 | 5.5 | 3.724 | 11.573 | 14.175 | 2.673 | 33.998 | 33.857 | 296.285 |
|  |  | 3.459 | 10.5 | 14.175 | 3.976 | 32.203 | 35.687 | 293.948 |
| 204.14 | 5.5 | 0.458 | 16.38 | 12.918 | 4.217 | 13.808 | 52.219 | 407.968 |
|  |  | 0.439 | 16.201 | 13.312 | 4.537 | 12.453 | 53.058 | 411.289 |
| 190 | 5.5 | 2.105 | 14.505 | 13.73 | 3.93 | 23.845 | 41.88 | 352.98 |
|  |  | 2.075 | 14.425 | 13.76 | 3.955 | 23.955 | 41.84 | 352.18 |
| 190 | 5.5 | 2.24 | 14.68 | 13.66 | 3.87 | 23.59 | 41.96 | 354.6 |
|  |  | 2.22 | 14.66 | 13.67 | 3.88 | 23.63 | 41.94 | 354.38 |
| 190 | 5.5 | 2.09 | 14.465 | 13.745 | 3.9425 | 23.9 | 41.86 | 352.54 |
|  |  | 1.96 | 14.25 | 13.83 | 4.014 | 24.207 | 41.74 | 350.73 |
| 190 | 7.62 | 1.613 | 15.093 | 13.593 | 3.936 | 18.281 | 47.484 | 380.145 |
|  |  | 1.239 | 15.045 | 13.768 | 4.096 | 20.979 | 44.873 | 369.969 |
| 190 | 3.38 | 2.449 | 13.857 | 14.637 | 3.459 | 24.385 | 41.213 | 348.113 |
|  |  | 2.158 | 14.026 | 13.218 | 3.994 | 25.788 | 40.816 | 342.37 |

**Data Used for Table 4. Antioxidant contents and activities of Sidama coffee within different roasting conditions**

| Run no | Temp  ^o^C | Time (min) | Phenol | Flavonoid | DPPH (µg/ml) | FRAP (mg/g) | EC_50_  (µg/ml) |
| --- | --- | --- | --- | --- | --- | --- | --- |
| 1 | 180 | 4 | 42.04 | 57.3 | 83.66 | 6.61 | 76.87 |
|  |  |  | 48.78 | 70.21 | 84.89 | 8.44 | 77.6 |
| 2 | 200 | 7 | 32.14 | 25.55 | 66.93 | 4.69 | 100.23 |
|  |  |  | 34.12 | 33.58 | 69.91 | 6.12 | 104.44 |
| 3 | 180 | 7 | 59.34 | 68.46 | 88.58 | 6.17 | 71.78 |
|  |  |  | 67.05 | 79.41 | 91.88 | 7.7 | 71.12 |
| 4 | 190 | 5.5 | 36.75 | 53.28 | 78.74 | 5.73 | 84.83 |
|  |  |  | 39.66 | 54.18 | 79.9 | 7.17 | 85.49 |
| 5 | 190 | 5.5 | 38 | 53.28 | 78.67 | 5.91 | 85 |
|  |  |  | 38 | 54.63 | 78.55 | 7.38 | 85 |
| 6 | 200 | 4 | 34.6 | 32.36 | 70.86 | 5.04 | 98.99 |
|  |  |  | 35.79 | 33.86 | 71.91 | 6.33 | 99.33 |
| 7 | 190 | 5.5 | 38.54 | 54.21 | 79.02 | 6.42 | 85.52 |
|  |  |  | 38.22 | 53.89 | 79.45 | 6.39 | 84.98 |
| 8 | 190 | 7.62 | 32.52 | 33.27 | 72.83 | 5.21 | 93.48 |
|  |  |  | 35.06 | 33.91 | 74.9 | 6.65 | 94.01 |
| 9 | 190 | 5.5 | 37.92 | 53.28 | 78.98 | 6.43 | 84.99 |
|  |  |  | 38.13 | 54.18 | 77.97 | 6.55 | 84.9 |
| 10 | 190 | 3.38 | 47.48 | 58.84 | 80.7 | 5.91 | 80.87 |
|  |  |  | 54.88 | 69.83 | 81.89 | 7.38 | 81.87 |
| 11 | 190 | 5.5 | 38.49 | 54.86 | 79.45 | 6.28 | 85.55 |
|  |  |  | 38 | 53.84 | 79.22 | 6.58 | 85.14 |
| 12 | 175.86 | 5.5 | 40.79 | 49.85 | 82.67 | 6.79 | 78.53 |
|  |  |  | 42.47 | 50 | 83.89 | 6.89 | 79.03 |
| 13 | 190 | 5.5 | 38.22 | 54.38 | 79.73 | 6.17 | 86.04 |
|  |  |  | 38.13 | 53.96 | 78.99 | 7.7 | 84.88 |
| 14 | 204.14 | 5.5 | 26.24 | 21.4 | 62.99 | 4.34 | 108.29 |
|  |  |  | 27.31 | 32.27 | 65.91 | 5.7 | 115.02 |

**Data used for Figures**

**Figure 1. Chromatograph representation of acrylamide for different roasting condition**

a) Light roasted coffee

Rep: - 1


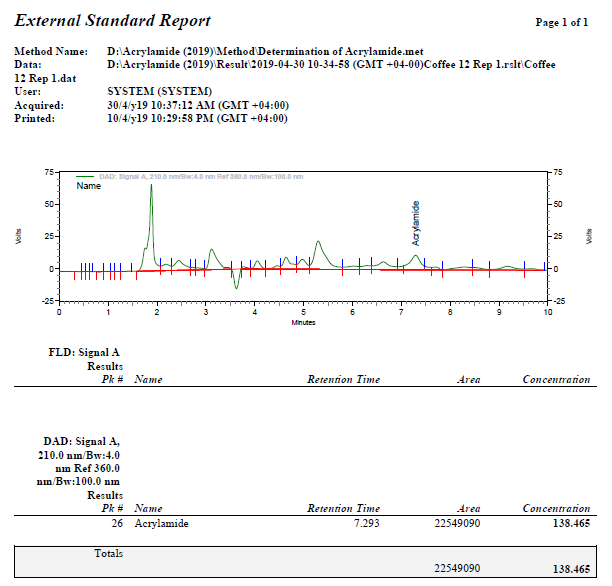


Rep: - 2


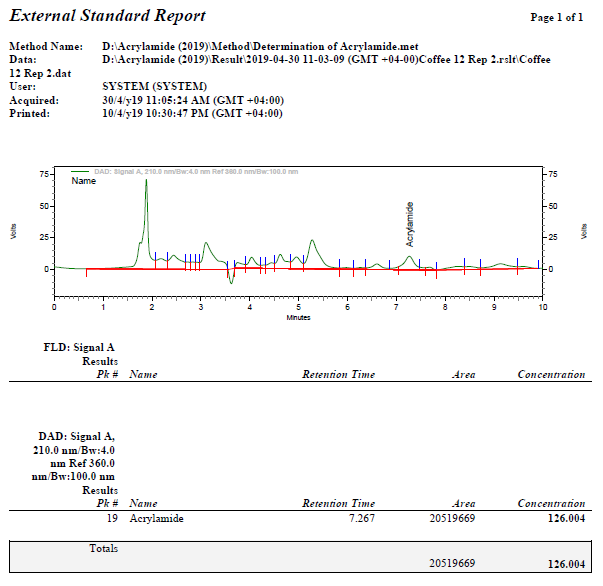


b) Medium roasted coffee

Rep: - 1


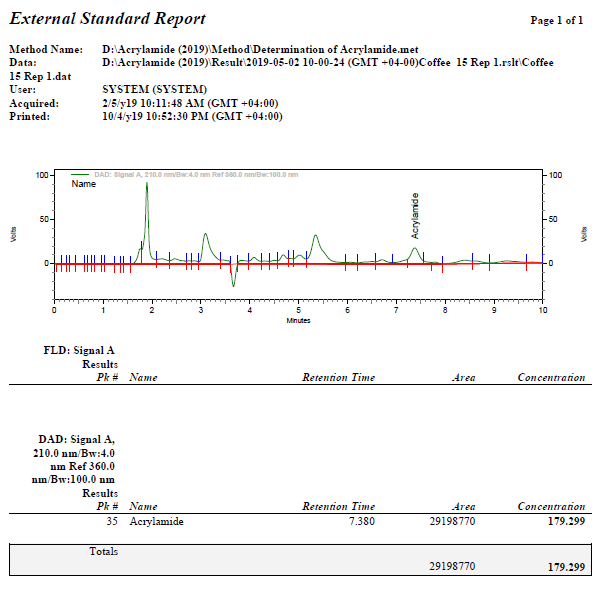


Rep: - 2


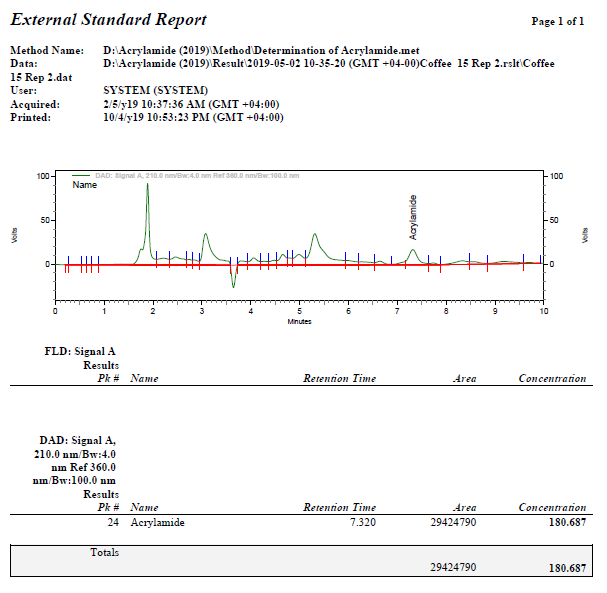


c) Medium dark roasted coffee

Rep: - 1


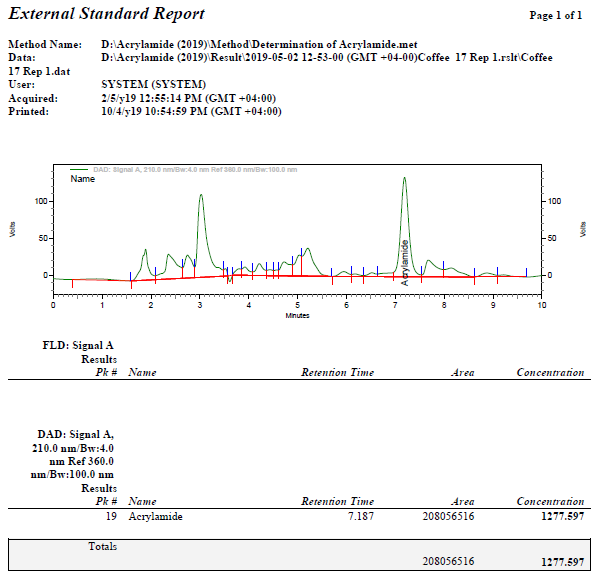


Rep: - 2


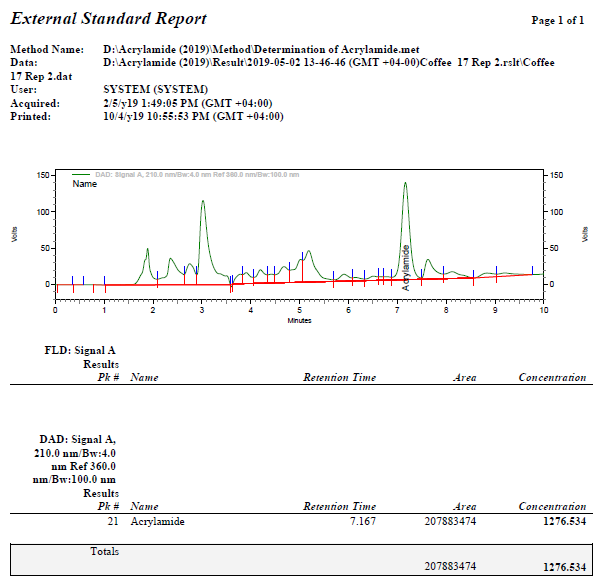


d) Dark roasted coffee

Rep: - 1


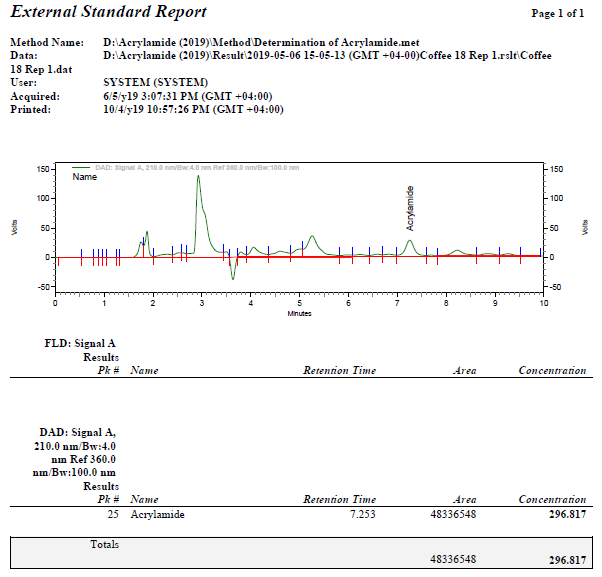


Rep: - 2


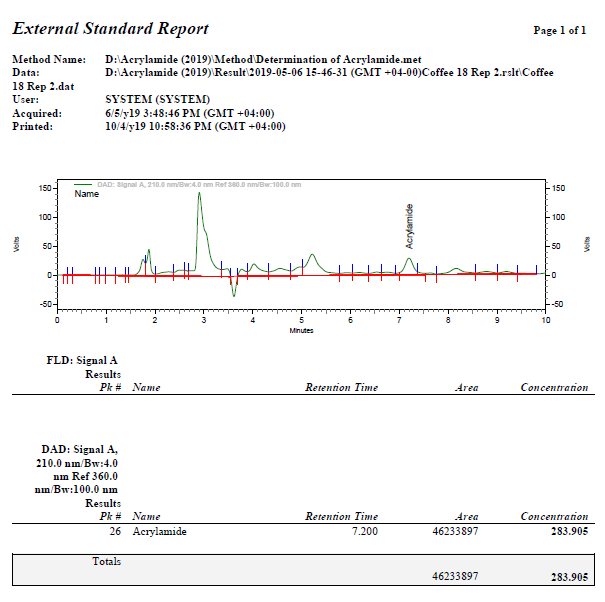


e) Standard


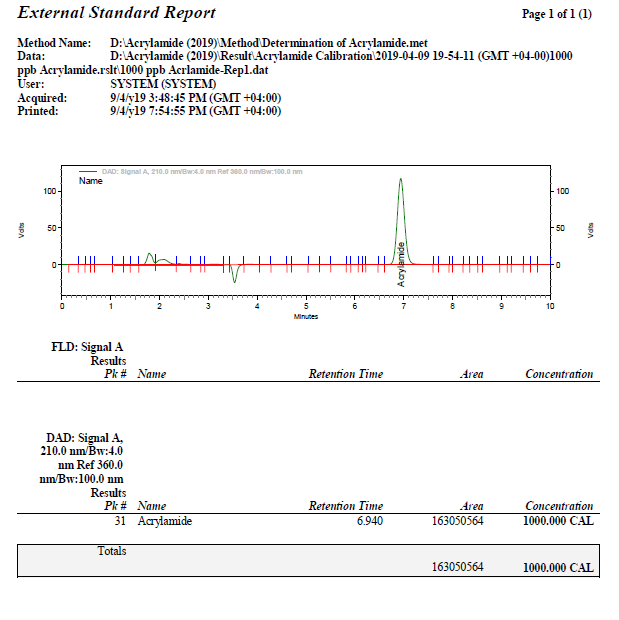


**Data used for Figure 2. 3D plot of a) acrylamide b) fat c) fiber d) protein content of optimized roasted Sidama coffee.**

| Run | Block | Temperature (^o^C) | Time (min) | Acrylamide (mg/kg) | Fat  (%) | Protein (%) | Fiber (%) |
| --- | --- | --- | --- | --- | --- | --- | --- |
| 1 | Block 1 | 190 | 5.5 | 0.36 | 14.53 | 13.72 | 23.82 |
| 2 | Block 1 | 200 | 7 | 0.577 | 17.27 | 13.55 | 15.094 |
| 3 | Block 1 | 180 | 7 | 0.281 | 12.94 | 14.04 | 27.45 |
| 4 | Block 1 | 190 | 5.5 | 0.359 | 14.42 | 13.76 | 23.95 |
| 5 | Block 1 | 190 | 5.5 | 0.358 | 14.5 | 13.73 | 23.84 |
| 6 | Block 1 | 180 | 4 | 0.264 | 12.93 | 14.16 | 30.12 |
| 7 | Block 1 | 200 | 4 | 1.988 | 15.23 | 13.54 | 18.32 |
| 8 | Block 2 | 175.86 | 5.5 | 0.202 | 11.036 | 14.18 | 33.1 |
| 9 | Block 2 | 204.14 | 5.5 | 0.512 | 16.29 | 13.12 | 13.13 |
| 10 | Block 2 | 190 | 5.5 | 0.359 | 14.46 | 13.74 | 23.9 |
| 11 | Block 2 | 190 | 5.5 | 0.36 | 14.67 | 13.66 | 23.61 |
| 12 | Block 2 | 190 | 5.5 | 0.358 | 14.36 | 13.79 | 24.053 |
| 13 | Block 2 | 190 | 7.62 | 2.549 | 15.07 | 13.68 | 19.63 |
| 14 | Block 2 | 190 | 3.38 | 0.344 | 13.94 | 13.93 | 25.086 |

**Data used for Figure 3. Antioxidant scavenging capacity of Sidama coffee**

| Con | STD | VL | L | LM | ML | M | MD | DM | D | VD |
| --- | --- | --- | --- | --- | --- | --- | --- | --- | --- | --- |
| 0 | 0 | 0 | 0 | 0 | 0 | 0 | 0 | 0 | 0 | 0 |
| 20 | 33.21 | 27.8964 | 28.2285 | 29.2248 | 27.2322 | 26.568 | 24.9075 | 23.9112 | 23.247 | 22.5828 |
| 40 | 40.82 | 33.8806 | 34.2888 | 35.5134 | 33.4724 | 31.4314 | 30.615 | 29.3904 | 28.574 | 24.492 |
| 80 | 80.45 | 67.578 | 68.3825 | 72.405 | 65.969 | 64.36 | 60.3375 | 57.924 | 56.315 | 52.2925 |
| 120 | 96.82 | 81.3288 | 82.297 | 84.2334 | 79.3924 | 77.456 | 72.615 | 69.7104 | 67.774 | 62.933 |
| 160 | 98.42 | 82.6728 | 83.657 | 88.578 | 80.7044 | 78.736 | 72.8308 | 70.8624 | 66.9256 | 62.9888 |
| 200 | 99.87 | 83.8908 | 84.8895 | 91.8804 | 81.8934 | 79.896 | 74.9025 | 71.9064 | 69.909 | 65.9142 |

LM=light medium; DM= medium light; M=medium; D=dark; L=light; MD=medium dark; VL=very light; ML=medium light and VD=very dark.

**Data used for Figure 4. Ferric reducing power of roasting Sidama coffee.**

| Con | STD | VL | L | LM | ML | M | MD | DM | D | VD |
| --- | --- | --- | --- | --- | --- | --- | --- | --- | --- | --- |
| 0 | 0 | 0 | 0 | 0 | 0 | 0 | 0 | 0 | 0 | 0 |
| 50 | 0.37532 | 0.315269 | 0.319022 | 0.307762 | 0.330282 | 0.300256 | 0.28149 | 0.27023 | 0.262724 | 0.255218 |
| 100 | 0.34292 | 0.284624 | 0.288053 | 0.281194 | 0.29834 | 0.264048 | 0.25719 | 0.246902 | 0.240044 | 0.205752 |
| 300 | 1.457 | 0.73522 | 0.8822 | 0.87692 | 0.94322 | 0.7122 | 0.602 | 0.442 | 0.4022 | 0.24702 |
| 500 | 1.60722 | 1.350065 | 1.366137 | 1.31792 | 1.398281 | 1.285776 | 1.205415 | 1.157198 | 1.125054 | 1.044693 |
| 800 | 2.00622 | 1.685225 | 1.705287 | 1.6451 | 1.805598 | 1.604976 | 1.484603 | 1.444478 | 1.36423 | 1.283981 |
| 1000 | 2.42032 | 2.033069 | 2.057272 | 1.984662 | 2.226694 | 1.936256 | 1.81524 | 1.74263 | 1.694224 | 1.597411 |
